# Toward Optimized Intravoxel Incoherent Motion (IVIM) and Compartmental T2 Mapping in Abdominal Organs

**DOI:** 10.1002/mrm.70278

**Published:** 2026-02-01

**Authors:** Julia Stabinska, Thomas A. Thiel, Hans‐Jörg Wittsack, Alexandra Ljimani, Helge J. Zöllner

**Affiliations:** ^1^ F.M. Kirby Research Center for Functional Brain Imaging, Kennedy Krieger Institute Baltimore Maryland USA; ^2^ Division of Nephrology, Department of Medicine The Johns Hopkins University School of Medicine Baltimore Maryland USA; ^3^ Medical Faculty, Department of Diagnostic and Interventional Radiology University Dusseldorf Dusseldorf Germany; ^4^ Division of MR Research, Russell H. Morgan Department of Radiology and Radiological Science The Johns Hopkins University School of Medicine Baltimore Maryland USA

**Keywords:** intravoxel incoherent motion, IVIM, liver, perfusion fraction, T2‐IVIM

## Abstract

**Purpose:**

To quantitatively assess the bias in the intravoxel incoherent motions (IVIM)–derived pseudo‐diffusion volume fraction (*f*) caused by the differences in relaxation times between the tissue and fluid compartments, and to develop a two‐dimensional (*b*‐value‐TE) fitting approach for simultaneous T2 and IVIM parameter estimation along with an optimal acquisition protocol for the relaxation‐compensated T2‐IVIM imaging in the liver.

**Methods:**

Simulations were conducted to investigate the TR‐ and TE‐dependent bias in *f* when using the IVIM model, and to evaluate the applicability of the 2D T2‐IVIM model for reducing this bias. The numerical findings were then validated using the in vivo IVIM data from four healthy volunteers on a 3‐Tesla MRI scanner. Finally, a numerical framework for optimizing the T2‐IVIM protocol for relaxation‐compensated *f* parameter estimation was proposed and tested using the in vivo data.

**Results:**

In vivo, the traditional IVIM model showed a trend toward higher *f* with increasing TE in the liver (*R* = 0.46, *p* = 0.023), but not in the kidney cortex (*R* = −0.067, *p* = 0.76) or medulla (*R* = 0.039, *p* = 0.86). In both simulations and in vivo, 2D T2‐IVIM modeling yielded lower *f* values and reduced variability in the liver. Our results further suggest that a *b*‐TE protocol with six *b*‐values and three TEs (50, 60, and 100 ms) may be optimal for liver T2‐IVIM.

**Conclusion:**

The extended 2D T2‐IVIM model effectively minimizes the TE‐dependent bias in *f* and allows simultaneous estimation of the IVIM parameter and compartmental T2 values in abdominal organs.

## 1 Introduction

The diffusion signal in biological tissues is influenced by both microstructure and microdynamics, including capillary blood flow, exchange, and transport between different tissue compartments [1]. Diffusion‐weighted MRI can, therefore, provide information on the hemodynamic status of tissues and detect changes in microvascular perfusion related to both physiological (e.g., response to stimuli or exercise [2, 3, 4]) and pathological conditions (e.g., stroke [5, 6], cancer [7, 8, 9, 10, 11], and kidney [12, 13, 14, 15, 16], and liver [17, 18, 19, 20] diseases).

As postulated by Le Bihan et al. in 1980s [21, 22], blood flow within isotropically distributed capillary segments induces phase dispersion of the MR signal, which results in enhanced signal attenuation in diffusion‐weighted images, especially at lower *b*‐values. To separate the contributions of “true” molecular diffusion and microcirculation of blood in the capillaries, they proposed a method called “intravoxel incoherent motion (IVIM) imaging” that relies on acquiring multi‐*b*‐value DWI data. This original IVIM model assumes that (i) the pseudo‐diffusion coefficient associated with microcirculation is substantially higher than the molecular diffusion coefficient, (ii) exchange between vascular and extracellular compartments is negligible during measurements, (iii) spins within the flowing blood change direction many times during the diffusion encoding period, due to the tortuous capillary network, and (iv) relaxation times of the involved compartments are identical. Under these conditions, the signal decay across different *b*‐values is expected to follow a biexponential function:

(1) $$\frac{S_{b}}{S_{0}}=\left(1-f\right)\cdot e^{-\mathrm{bD}}+f\cdot e^{-bD^{*}}$$

where *f* is the pseudo‐diffusion volume fraction (commonly referred to as the perfusion fraction), *D* and D* are the “true” and pseudo‐diffusion coefficients, and *S*_*b*_ and *S*_0_ are the signal intensities measured with gradient sensitivity factor *b* and *b* = 0, respectively.

IVIM data are typically acquired with a single echo time, usually set to the minimum value allowed by the sequence, which is determined by the time needed to accommodate the longest diffusion gradient required for the highest *b*‐value, along with the spin‐refocusing pulse and imaging readout. The TE and TR dependencies are then generally incorporated into the *S*_0_ term during the data fitting step. However, since both IVIM model compartments exhibit distinct and variable T1 and T2 relaxation times, the traditional IVIM model is prone to inaccuracies in estimating the pseudo‐diffusion volume fraction [22]. In particular, as body fluids such as blood and pre‐urine are known to have longer T2 than most tissues [23], *f* tends to be overestimated as a function of increasing TE [24], by a factor proportional to the difference in T2 between the tissue and fluid components [22]. If unaccounted for, the TE‐dependence of *f* may lead to substantial differences in volume fraction estimates across sites and/or scanners in clinical trials, thereby increasing variability and reducing reproducibility.

In the context of standardizing the use of IVIM in clinical studies, the primary contributions of this study are: (i) a quantitative assessment of how relaxation time differences between tissue and flow‐related compartments influence IVIM parameter estimation, (ii) the development of a two‐dimensional (2D) IVIM data fitting approach for simultaneous estimation of the IVIM parameters and compartmental T2 values, and (iii) the introduction of a numerical framework for optimizing the *b*‐TE protocol for IVIM imaging. Our findings are validated using in vivo IVIM data collected in the kidneys and liver of healthy volunteers on a clinical 3 T MRI system.

## 2 Methods

In silico DW signal generation, data fitting, analysis, and visualization were performed using in‐house developed scripts in MATLAB (2024b, Mathworks, MA) and Python 3.10 and are freely available on Github: https://github.com/stabinska/T2‐IVIM.

### 2.1 Numerical Phantom

Diffusion‐weighted MR signals were generated using the modified relaxation‐compensated IVIM equation:

(2) $$\frac{S}{S_{0}}=\frac{\begin{array}{c} \left(1-f\right)\cdot \left(\left(1-\exp\left(-\frac{\mathrm{TR}}{T1_{\text{tissue}}}\right)\right)\cdot \exp\left(-\frac{\mathrm{TE}}{T2_{\text{tissue}}}-\mathrm{bD}\right)\right)\\ +\;f\cdot \left(\left(1-\exp\left(-\frac{\mathrm{TR}}{T1_{\text{fluid}}}\right)\right)\cdot \exp\left(-\frac{\mathrm{TE}}{T2_{\text{fluid}}}-bD^{*}\right)\right) \end{array}}{\begin{array}{c} \left(1-f\right)\cdot \exp\left(-\frac{\mathrm{TE}}{T2_{\text{tissue}}}\right)\cdot \left(1-\exp\left(-\frac{\mathrm{TR}}{T1_{\text{tissue}}}\right)\right)\\ +\;f\cdot \exp\left(-\frac{\mathrm{TE}}{T2_{\text{fluid}}}\right)\cdot \left(1-\exp\left(-\frac{\mathrm{TR}}{T1_{\text{fluid}}}\right)\right) \end{array}}$$

where T1_tissue_, T2_tissue_ and T1_fluid_, T2_fluid_ are the longitudinal and transverse relaxation times for the tissue and fluid (e.g., blood and/or pre‐urine in the kidney) compartments, respectively. The DW signals were generated using biologically realistic estimates of IVIM parameter values and different ranges of T1 and T2 relaxation times at 3T for the kidney and liver. A multi‐*b*‐value acquisition protocol with 16 *b*‐values: (0, 10, 20, 30, 50, 70, 100, 150, 200, 250, 300, 350, 450, 550, 650, and 750s^2^/mm), 6 TE values (47–72 ms in 5 ms steps) and 5 TR values (2000–4000 ms in 500 ms steps) were chosen. Monte‐Carlo simulations were performed by generating 2500 repetitions of each parameter combination with 6 different levels of Rician noise SD: 0, 0.005, 0.010, 0.015, 0.02, 0.025, and 0.03, corresponding to the SNR levels of 200, 100, 66, 50, 40, and 33, respectively. A summary of all simulation parameters for the kidney and liver phantoms is given in Table 1.

**TABLE 1:** Tissue and acquisition parameters used for numerical simulations.

Parameter	Value	
Phantom type	Kidney at 3T	Liver at 3T
*f* (%)	15%	9.5%
*D* (mm^2^/s)	0.0016	0.0010
D* (mm^2^/s)	0.012	0.067
T1_tissue_ (ms)	1200	800
T1_fluid_ (ms)	1200–1800 in 50 ms steps	800–1400 in 50 ms steps
T2_tissue_ (ms)	67	27
T2_fluid_ (ms)	67–150 in 15 ms steps	27–87 in 5 ms steps
*b*‐values (s/mm^2^)	*b*‐values set 1: 0, 10, 20, 30, 50, 70, 100, 150, 200, 250, 300, 350, 450, 550, 650, 750 *b*‐values set 2: 0, 10, 100, 200, 500, and 800	*b*‐values set 1: 0, 10, 20, 30, 50, 70, 100, 150, 200, 250, 300, 350, 450, 550, 650, 750 *b*‐values set 2: 0, 10, 20, 100, 200, and 550
TE (ms)	TE set 1: 47–72 in 5 ms steps TE set 2: 50–100 in 5 ms steps	TE set 1: 47–72 in 5 ms steps TE set 2: 50–100 in 5 ms steps
TR (ms)	2000, 2500, 3000, 3500, 4000	2000, 2500, 3000, 3500, 4000
SD_Rician noise_/SNR at *S*_0_ (SNR_*S*0_)	0/∞, 0.005/200, 0.01/100, 0.015/75, 0.02/66, 0.025/40, and 0.03/33	0/∞, 0.005/200, 0.01/100, 0.015/75, 0.02/66, 0.025/40, and 0.03/33
Repetitions (*N*_rep_)	2500	2500

### 2.2 IVIM Model Fitting

**FIGURE 1:**
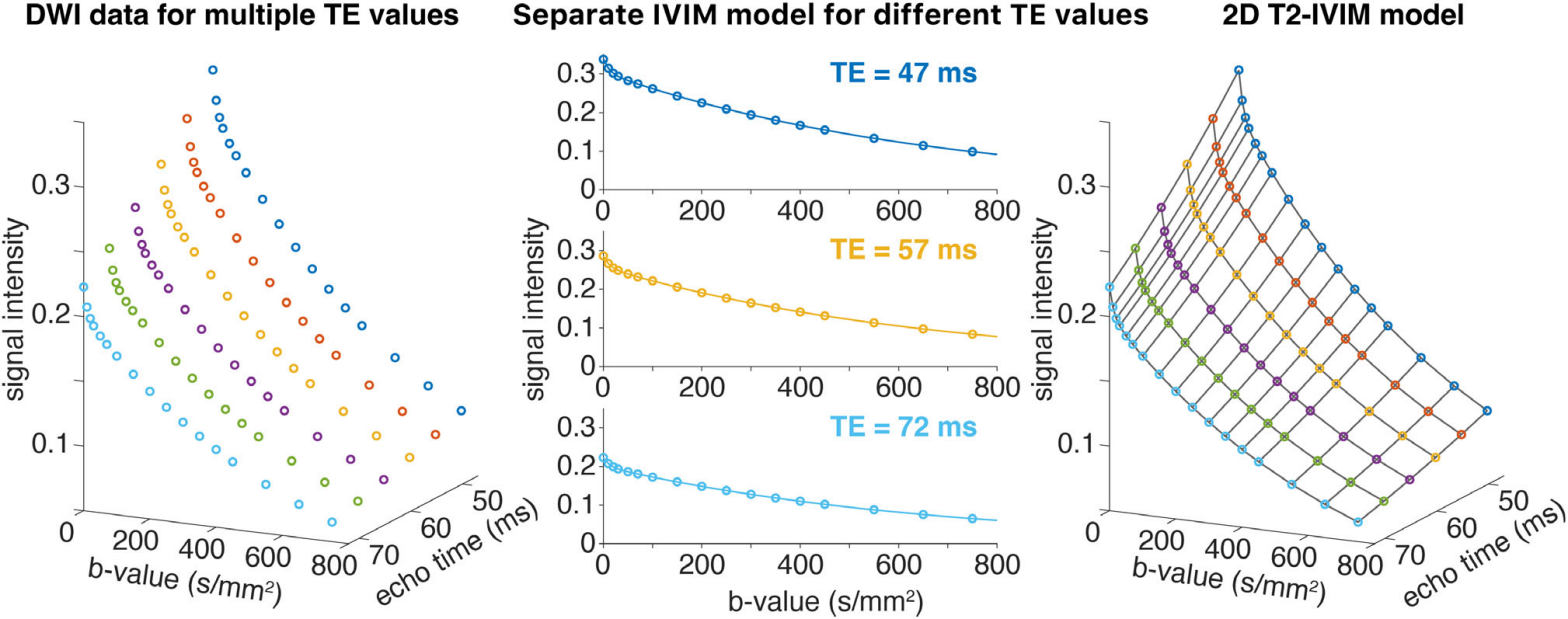
A schematic diagram of data modeling approaches used in this study. DW signals at different TE (and TR), *b*‐values, and noise levels were generated (left panels) using Equation (1) with realistic relaxation times at 3T for the liver and kidney (Table 1). These signals were then fitted using the conventional IVIM model for each TE separately (middle panel—only three exemplary TEs), and the extended 2D T2‐IVIM modeling for all combinations of TE and *b*‐values. Multi‐*b*‐value (Table 1, *b*‐values set 1) DW signals without noise at six different TE values (Table 1, TE set 1) and a single TR = 3000 ms are depicted for visualization purposes.

The generated DW signals were normalized to S_0_ signal corresponding to b = 0, TR = TR′, and TE = TE′ and subsequently fitted using the conventional IVIM model (Equation 1) for each TE separately to determine the IVIM parameters (*D*, D*, and *f*) (*1D IVIM fit*), and the extended 2D T2‐IVIM modeling (*2D T2‐IVIM fit*) simultaneously including all 6 TE values and all 16 *b*‐values to estimate the IVIM parameters as well as the T2_tissue_ and T2_fluid_ values (Figure 1) using a T2‐relaxation compensated IVIM equation (excluding T1 terms). The IVIM parameters and compartmental T2 values were determined simultaneously by reparametrizing the 2D data and modeling as a one‐dimensional (1D) problem (i.e., the 2D *b*‐TE data are “flattened” by sequentially concatenating all *b*‐value data across increasing TE values to create a 1D dataset for fitting). Both the 1D and 2D models were fitted using a one‐step nonlinear least‐squares approach based on a trust‐region algorithm. The bias (in %) in the IVIM parameter estimates was calculated as 100⋅(*f*_fit_ − *f*_true_)/*f*_true_. Variability across the 2500 repetitions was assessed using the mean and standard deviation.

### 2.3 In Vivo MRI Experiments

The study was conducted according to the guidelines of the Declaration of Helsinki and approved by the Ethics Committee of the Medical Faculty, University of Dusseldorf, Germany. Written consent was obtained from each participant before the MRI examination. Four healthy subjects (three females and one male; age range: 25–30 years; mean age: 26.5 ± 3.1 years) with no history of renal or liver diseases were included in this study. Subjects were not given any restrictions regarding fluid or food intake.

MRI examinations were conducted on a 3 MRI system (MAGNETOM Prisma; Siemens Heathineers, Erlangen, Germany) using an 18‐channel torso array coil and a 32‐spine coils. For anatomical visualization and planning, T2 HASTE (half Fourier single‐shot turbo spin‐echo) coronal oblique images were acquired. DWI data were collected using a DW monopolar pulsed gradient spin‐echo (PGSE) sequence with the following parameters: TR = 1900 ms; field of view (FOV): 370 × 370 mm^2^; acquisition matrix: 176 × 176; voxel size: 2.1 × 2.1 × 5.0 mm^3^; bandwidth: 1894 Hz/Px, GRAPPA factor: 2; Phase Partial Fourier: 5/8; 3 slices in oblique coronal plane; 3 diffusion directions (3D diagonal); *b*‐values (averages): 0 (3), 10 (3), 20 (3), 30 (3), 50 (3), 70 (4), 100 (4), 150 (4), 200 (4), 250 (4), 300 (4), 350 (4), 450 (5), 550 (5), 650 (5), and 750 (5) s/mm^2^, and spectral‐attenuated inversion‐recovery fat saturation for fat suppression. This sequence was repeated at six different TE values = 47, 52, 57, 62, 67, and 72 ms. The diffusion time (Δ = 20 ms) and diffusion gradients duration (*δ* = 6.2 ms) were kept constant at each TE to minimize their influence on the IVIM parameter [25]. The DWI acquisition was respiratory‐triggered at the exhale phase of the respiratory cycle with a 20% threshold. Depending on the subject's respiration rate, the average acquisition time for each DWI acquisition was about 6–8 min.

### 2.4 In Vivo Data Analysis

Retrospective 2D registration based on a normalized mutual information similarity measure was performed to align all DW images acquired at different TE‐ and *b*‐values using an in‐house‐developed software based on ANTs (http://stnava.github.io/ANTs). The whole FOV was used for spatial registration. After the motion correction, a 3 × 3 Gaussian filter was applied to each image. Following a visual inspection by an experienced abdominal radiologist (A.L., 10 years of experience), a single slice that was least affected by respiratory motion and contained clearly visible kidney cortex, kidney medulla, and liver in *S*_0_ image was selected for manual ROI segmentation in ITK‐SNAP [26] (version 3.8.0) and further analysis. For the liver, all visible parenchymal regions were segmented while carefully excluding major blood vessels and bile ducts.

The in vivo data were fitted pixel‐wise using the traditional IVIM model for each TE separately and using the 2D T2‐IVIM model. In contrast to the numerical phantom, which included both T1 and T2 terms to assess relaxation effects, the in vivo 2D T2‐IVIM fitting was performed using a T2‐relaxation–compensated IVIM model without explicit T1 fitting. This choice was motivated by simulation results showing that T1 effects are less pronounced and can be mitigated by appropriate TR selection. Similarly, a T2‐compensated *S*_0_ was estimated as a free parameter in the in vivo fitting, whereas this was not the case in the numerical experiments. To improve the robustness of compartmental T2 and IVIM parameter estimation, a modified version of the Image Downsampling Expedited Adaptive Least‐Squares (IDEAL) [27, 28] approaches was applied. Eleven downsampling steps were applied (1 × 1, 2 × 2, 4 × 4, 8 × 8, 16 × 16, 32 × 32, 64 × 64, 96 × 96, 128 × 128, 152 × 152, and 176 × 176) and the initial guesses and boundaries for each fitting step were constrained to 50% of the initial values for *S*_0_, and 20% for *f*, *D*, D*, and T2_tissue_ and T2_fluid_ (for the 2D fit). The initial parameter values in each iterative step were determined by spatially interpolating the estimated parameters from the prior downsampled image using bilinear interpolation.

### 2.5 Numerical TE‐*b* Protocol Optimization and in Vivo Validation

Based on the results from our in vivo study, the TE‐b protocol optimization for T2‐IVIM imaging was performed only for the liver. Four different combinations of *b*‐values and TE values were considered for optimization: (1) 16 *b*‐values (*b*‐values set 1) and 6 TE values (47–72 ms in 5 ms steps, TE set 1)—the “in vivo protocol”; (2) 16 *b*‐values (*b*‐values set 1) and 11 TE values (50–100 ms in 5 ms steps, TE set 2); (3) 6 “consensus” *b*‐values (*b*‐values set 2) and 6 TE values (TE set 1); and (4) 6 “consensus” *b*‐values (*b*‐values set 2) and 11 TE values (TE set 2). The “consensus” *b*‐values were derived from a Cramér–Rao Lower Bound analysis under the assumption of Gaussian noise [29, 30]. This work was conducted as part of broader IVIM community initiative aimed at standardizing acquisition protocols, discussed during a recent ISMRM workshop [31].

The optimal sets of TE values for the relaxation‐compensated *f* estimation were found using a genetic algorithm solver (*ga* function in the Global Optimization Toolbox, MATLAB), which is a stochastic, population‐based algorithm for solving both constrained and unconstrained optimization problems that is based on a natural selection process. In each generation, the genetic algorithm selects a set of individuals from the current population to be “parents” and uses them to produce “children” for the next generation. Over successive generations, the population “evolves” toward an optimal solution through mutations and crossover among population members [32]. The genetic algorithm offers several advantages over the least‐squares solvers, particularly in complex and nonlinear optimization problems, including higher robustness against finding local minima in large search spaces [33]. The genetic algorithm was used to minimize the normalized root‐mean‐square‐error (nRMSE) of the pseudo‐diffusion volume *f*:

(3) $$n\text{RMSE}=\sum_{i=1}^{N_{T2_{\text{fluid}}}}\frac{\sqrt{\beta_{f_{i}}^{2}+\sigma_{f_{i}}^{2}}}{f_{i}}$$

with the bias *β* and standard deviation *σ* for a given pseudo‐diffusion volume fraction, *f*_*i*_ summed across the different T2_fluid_ values. The normalization ensures that relative deviations not absolute ones are considered. This way the coefficient of variation is minimized instead of the absolute standard deviation of *f*. The variance was estimated using the Cramer–Rao Lower Bound (CRLB) of the pseudo‐diffusion fraction *f* to improve the speed of the optimization process. The optimization was repeated to find the optimal TE values for a 2, 3, 4, and 5 TEs TE‐*b* protocol for all four combinations of *b*‐ and TE‐values. For the genetic algorithm the population size was set to 10 000 with 100 total generations and a function tolerance of 0.001. The TE values from the fittest 5% of the population were preserved between generations. To evaluate the results of the protocol optimization, Monte‐Carlo simulations were performed with *N*_rep_ = 2500 repetitions and SNR_S0_ = 40. The TR was kept constant at 4000 ms. All parameters used for the liver phantom used for protocol optimization can be found in Table 1. Variability across the 2500 repetitions was evaluated by calculating the mean and standard deviation. Note that while the focus of this manuscript was on estimating *f*, the provided framework also enables optimization of other IVIM and T2 parameters, as well as their combinations.

Results of the numerical protocol optimization were then tested for the liver using all 16 *b*‐values (*b*‐values set 1) and different sets of the optimal TE values from the TE set 1. To evaluate the relative bias in *f*, a difference Δ*f* map was calculated by subtracting the *f* map obtained with each TE‐*b* protocol from the “ground truth” *f* map obtained using the full TE‐*b* protocol (6 TEs and 16 *b*‐values).

### 2.6 Statistical Analysis

For the Monte‐Carlo simulations, a one‐sided *F*‐test was used to assess whether the standard deviation of the *f* estimates across 2500 repetitions from the 2D T2‐IVIM fit is smaller than that from the conventional IVIM fit, and to compare the distributions of *f* values obtained with different optimized protocols. To quantify the size of the effect beyond statistical significance, the relative (percent) difference in standard deviation between methods was reported as an effect size metric: Δ*σ* (%) = 100 × (*σ*_1_ − *σ*_2_)/*σ*_2_, where *σ*_*i*_ is the standard deviation of the *f* estimates obtained with the method *i*.

Mean values of the in vivo IVIM parameter estimates within the ROIs obtained from the 2D T2‐IVIM and conventional IVIM modeling for each TE value were compared using planned pairwise ANOVA comparisons with Bonferroni correction with an adjusted significance level of *α* = 0.00833 (=0.05/6). The relationship between the IVIM parameters and TE from the conventional IVIM modeling was assessed using the Spearman correlation with a significance level *α* = 0.05.

## 3 Results

### 3.1 Effects of Relaxation Time Differences Between the IVIM Compartments on Parameter Estimation

**FIGURE 2:**
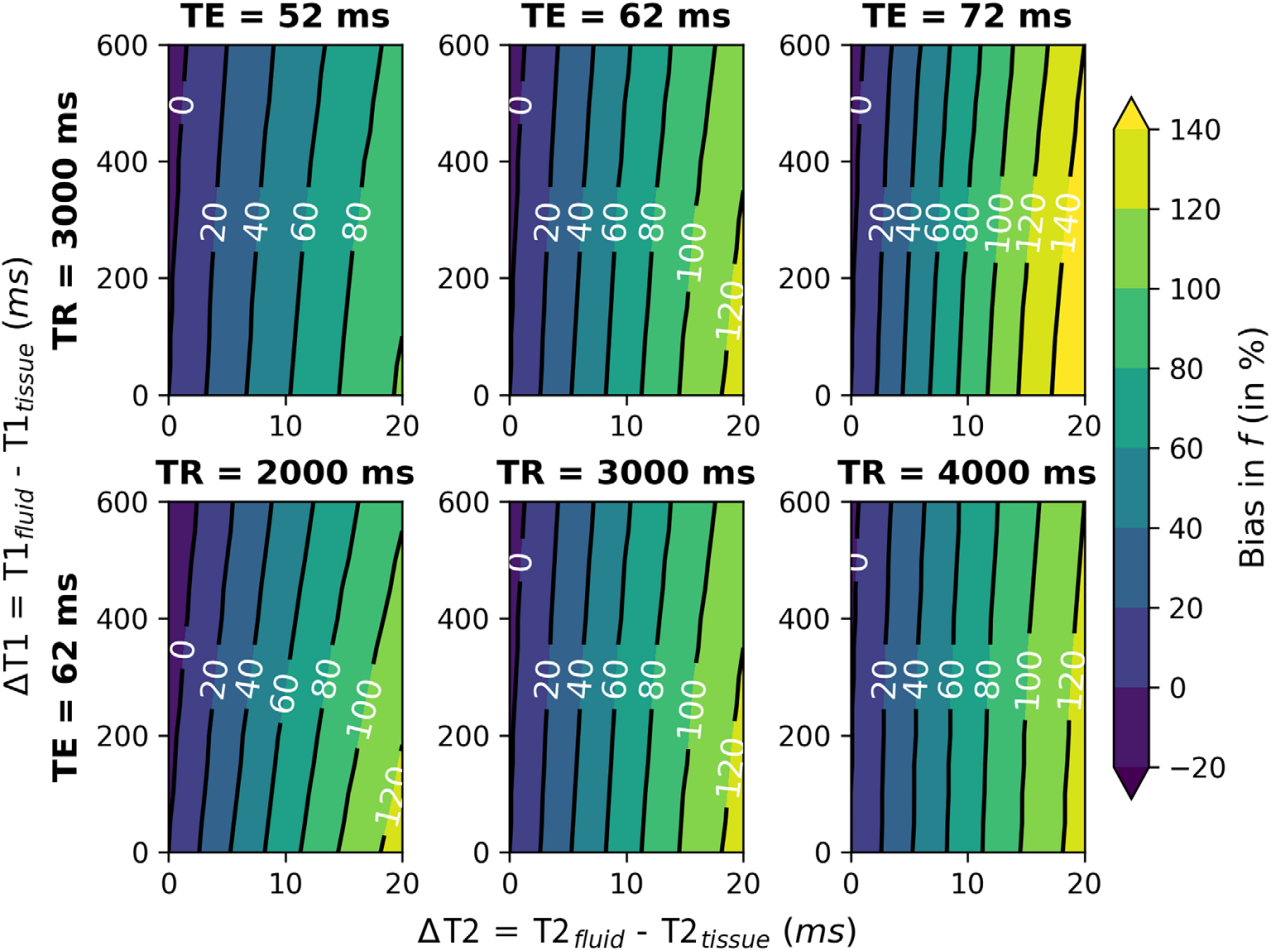
A contour plot of bias in pseudo‐diffusion volume fraction *f* (%) calculated using simulated liver data (Table 1, TE set 1 and *b*‐values set 1) as a function of differences in compartmental relaxation times (ΔT1 = T1_fluid_ − T1_tissue_ and ΔT2 = T2_fluid_ − T2_tissue_) at various TE (52, 62, and 72 ms) and TR (2000, 3000, and 4000 ms) generated without Rician noise. The bias was calculated as 100 ×(*f*_fit_ − *f*_true_)/*f*_true_. While the effect of ΔT1 on f parameter estimation is minimal, ΔT2 ∼ 5 ms leads to a 20% bias at TE = 52 ms and > 40% bias at TE = 72 ms.

As shown in Figure 2, pseudo‐diffusion volume fraction *f* in the liver strongly depends on the differences in relaxation times (ΔT1 = T1_fluid_ − T1_tissue_ and ΔT2 = T2_fluid_ − T2_tissue_) between both compartments, and thus on selected TE and TR. In fact, even at a relatively short TE of 52 ms, the bias in *f* exceeds 20% for ΔT2 as small as 5 ms. The effect of differences in compartmental T1 on the accuracy of *f* parameter estimation is less pronounced and can be minimized by choosing a longer TR (≥ 3000 ms). Figure S1 shows the results for the kidney, where 20% bias in *f* was found for ΔT2 higher than 20 ms. As expected, *D* and D* were not affected by the differences in compartmental relaxation times for neither organ.

**FIGURE 3:**
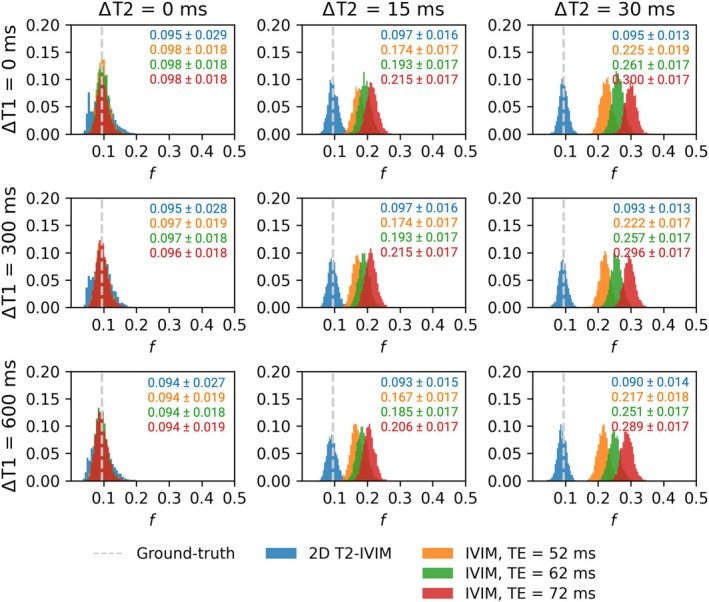
Distributions of pseudo‐diffusion volume fraction f obtained from 2D T2‐IVIM modeling (blue) and conventional IVIM modeling at TE = 52 ms (yellow), TE = 62 ms (green), and TE = 72 ms (red) using the simulated liver data (Table 1, TE set 1, *b*‐values set 1) for various ΔT1 = T1_fluid_ − T1_tissue_ and ΔT2 = T2_fluid_ − T2_tissue_. The gray dotted line represents the “ground truth” *f*_true_ value. Monte‐Carlo simulations were performed with *N*_rep_ = 2500 repetitions and SNR_S0_ = 40. The TR was kept constant at 4000 ms. The 2D T2‐IVIM fitting produced accurate *f* estimates over a wide range of ΔT1 and ΔT2 values. Mean ± SD across 2500 repetitions is reported for each distribution.

As demonstrated in Figure 3, an extended 2D T2‐IVIM model that allow for distinct T2 values in both compartments can be used to efficiently minimize the bias in *f* estimates for ΔT2 > 30 ms and over a wide range of ΔT1 values (> 600 ms) in the liver. For all ΔT2 > 0, the distributions obtained with 2D T2‐IVIM fitting are notably sharper than those from conventional IVIM fitting, as reflected by significantly lower standard deviations (*p* < 0.0001 for all comparisons), with relative (percent) reductions in standard deviation ranging from 9% to 38%. Similar results were observed in the kidney (Figure S2), where 2D fitting yielded significantly lower variability (*p* < 0.0001 for all comparisons), with relative reductions in standard deviation ranging from 44% to 94%, while only marginally improving the accuracy of f estimation.

As shown in Figures S3 and S4, 2D T2‐IVIM fitting provided accurate *f* estimates over a wide range of SNR levels. Although the variability of the *f* estimates increased at lower SNR, the standard deviation of the distributions from the 2D fit was significantly lower than that from the conventional 1D IVIM fit (*p* < 0.0001 for all comparisons), with relative reductions in standard deviation ranging from 6% to 16%. As demonstrated in Figure S4, D* showed reduced accuracy and increased variability at lower SNR (D* bias of 18% at SNR_S0_ = 40) for the conventional IVIM model, but not for the 2D T2‐IVIM model.

### 3.2 In Vivo Application of 2D T2‐IVIM Modeling in the Liver and Kidney

**FIGURE 4:**
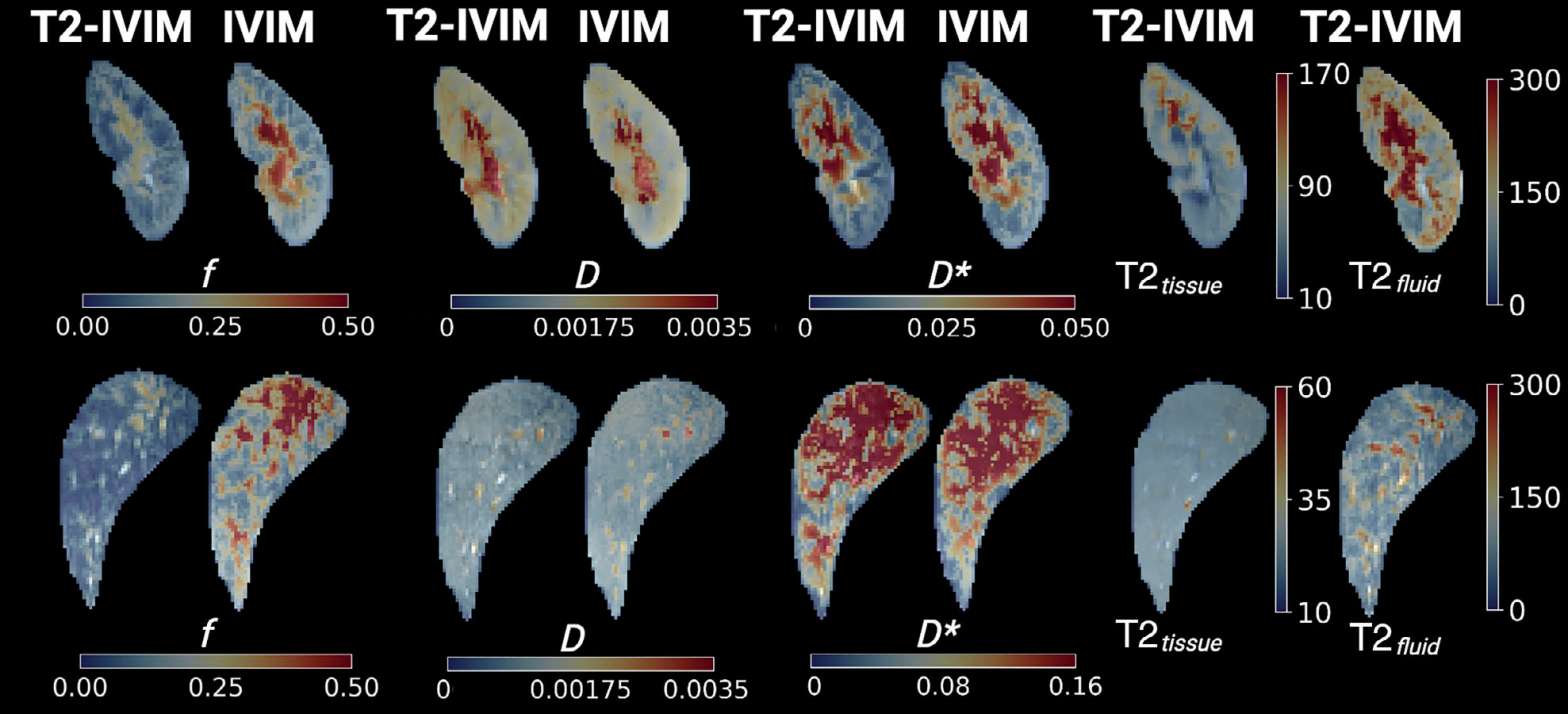
Representative examples of parameter maps obtained in the kidney and liver of a healthy volunteer using the conventional IVIM (TE = 47 ms) and 2D T2‐IVIM modeling. The f maps obtained from the 2D fitting show lower values than those from the conventional IVIM fitting in regions with high flow volume fractions, both in the liver and kidney.

Figure 4 shows representative examples of the T2 and IVIM parameter maps in the kidney and liver of a healthy volunteer obtained from the data fit to the conventional IVIM model (at TE = 47 ms) and 2D T2‐IVIM model with the IDEAL approach. The pseudo‐diffusion volume fraction maps show clear differences between the IVIM and T2‐IVIM models with overall higher *f* values in regions with high flow fractions, like the renal pelvis and the liver. As expected, the differences in the diffusion coefficient and pseudo‐diffusion coefficient maps between the 1D and 2D models are less pronounced than those in the *f* maps.

**FIGURE 5:**
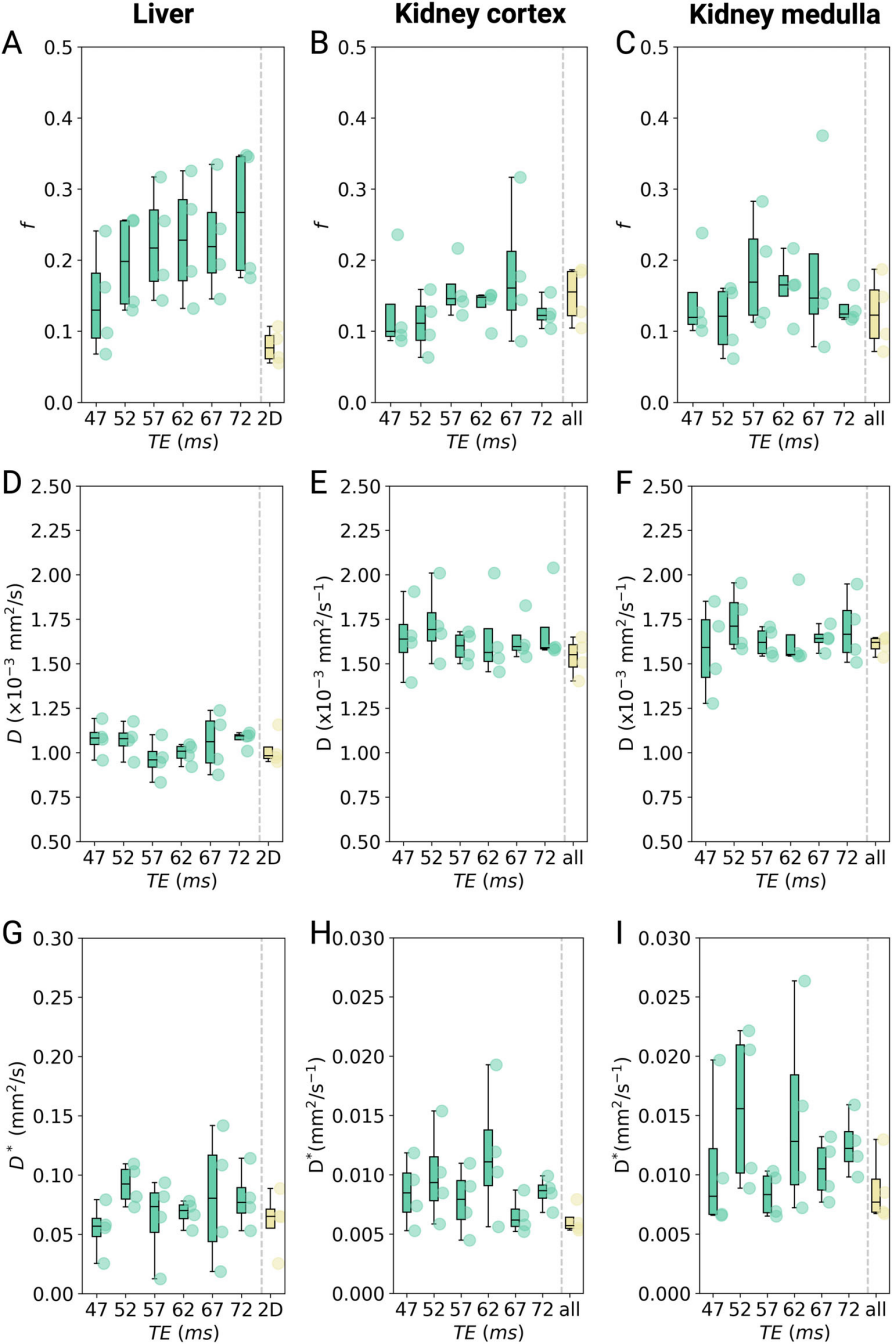
Boxplots for all IVIM parameters (*N* = 4) in the liver, kidney cortex, and kidney medulla for IVIM fitting per TE (green) and simultaneous 2D T2‐IVIM fitting (yellow). Circles indicate individual volunteers. f increases with TE in the liver but shows no TE dependence in the kidney. The 2D T2‐IVIM approach yields lower f than conventional IVIM modeling in the liver, but not in the kidney. *p < 0.0083. *D*, diffusion coefficient; D*, pseudo‐diffusion coefficient; *f*, pseudo‐diffusion volume fraction.

The results of the ROI‐based group analysis are demonstrated in Figure 5. A trend toward higher *f* with longer TE can be observed in the liver for the traditional IVIM model (*R* = 0.46, *p* = 0.023), which is consistent with our numerical findings. While generally not statistically significant (*p* > 0.0083 for all comparisons), this effect appears to be largely reduced when 2D T2‐IVIM modeling was applied, leading to overall decreased *f* values and lower variability between the subjects compared to the IVIM modeling. Interestingly, no such trend was observed in the kidney in the considered TE values range (*p* > 0.17 for all comparisons). Furthermore, no significant correlations between *f* and TE were found neither in the kidney cortex (*R* = −0.067, *p* = 0.76) nor kidney medulla (*R* = 0.039, *p* = 0.86). Similarly, there was no difference in *D* and D* obtained from the 2D T2‐IVIM and IVIM fitting (*p* > 0.05 for all comparisons) and no significant correlations between these parameters and TE (all *p* > 0.05) in neither organ (Figure 5B,C). Table 2 shows the mean and standard deviation values for the VIM and compartmental T2 parameter estimates in the liver and kidney, respectively.

**TABLE 2:** Mean and standard deviation of compartmental T2 and IVIM parameter estimates in the liver, kidney cortex, and kidney medulla.

Parameter	2D	TE = 47 ms	TE = 52 ms	TE = 57 ms	TE = 62 ms	TE = 67 ms	TE = 72 ms
Liver							
*f*	0.08 ± 0.02	0.14 ± 0.08	0.20 ± 0.07	0.22 ± 0.08	0.23 ± 0.09	0.23 ± 0.08	0.26 ± 0.10
*D* (mm^2^/s × 10 ^−3^)	1.02 ± 0.10	1.08 ± 0.10	1.07 ± 0.10	0.96 ± 0.11	1.00 ± 0.06	1.06 ± 0.17	1.08 ± 0.05
D* (mm^2^/s × 10 ^−3^)	61 ± 26	54 ± 22	92 ± 17	63 ± 36	68 ± 11	80 ± 55	80 ± 25
T2_tissue_ (ms)	26 ± 2						
T2_fluid_ (ms)	57 ± 8						
Cortex							
*f*	0.15 ± 0.04	0.13 ± 0.07	0.11 ± 0.04	0.15 ± 0.05	0.14 ± 0.03	0.18 ± 0.1	0.13 ± 0.02
*D* (mm^2^/s × 10 ^−3^)	1.54 ± 0.1	1.64 ± 0.18	1.72 ± 0.18	1.59 ± 0.07	1.64 ± 0.21	1.63 ± 0.11	1.69 ± 0.19
D* (mm^2^/s × 10 ^−3^)	6.2 ± 1.2	8.5 ± 2.8	9.9 ± 4.0	7.2 ± 2.0	12 ± 5.7	6.5 ± 1.5	8.5 ± 1.3
T2_tissue_ (ms)	63 ± 14						
T2_fluid_ (ms)	159 ± 59						
Medulla							
*f*	0.13 ± 0.05	0.14 ± 0.06	0.12 ± 0.05	0.17 ± 0.09	0.16 ± 0.05	0.19 ± 0.13	0.13 ± 0.02
*D* (mm^2^/s × 10 ^−3^)	1.61 ± 0.05	1.58 ± 0.22	1.74 ± 0.15	1.63 ± 0.07	1.66 ± 0.18	1.64 ± 0.06	1.70 ± 0.17
D* (mm^2^/s × 10 ^−3^)	8.8 ± 2.9	10.7 ± 6.2	15.5 ± 6.8	8.0 ± 1.6	14.7 ± 8.5	10.5 ± 2.5	12.5 ± 2.6
T2_tissue_ (ms)	71 ± 21						
T2_fluid_ (ms)	154 ± 44						

### 3.3 Numerical Protocol Optimization for 2D T2‐IVIM Imaging of the Liver

**FIGURE 6:**
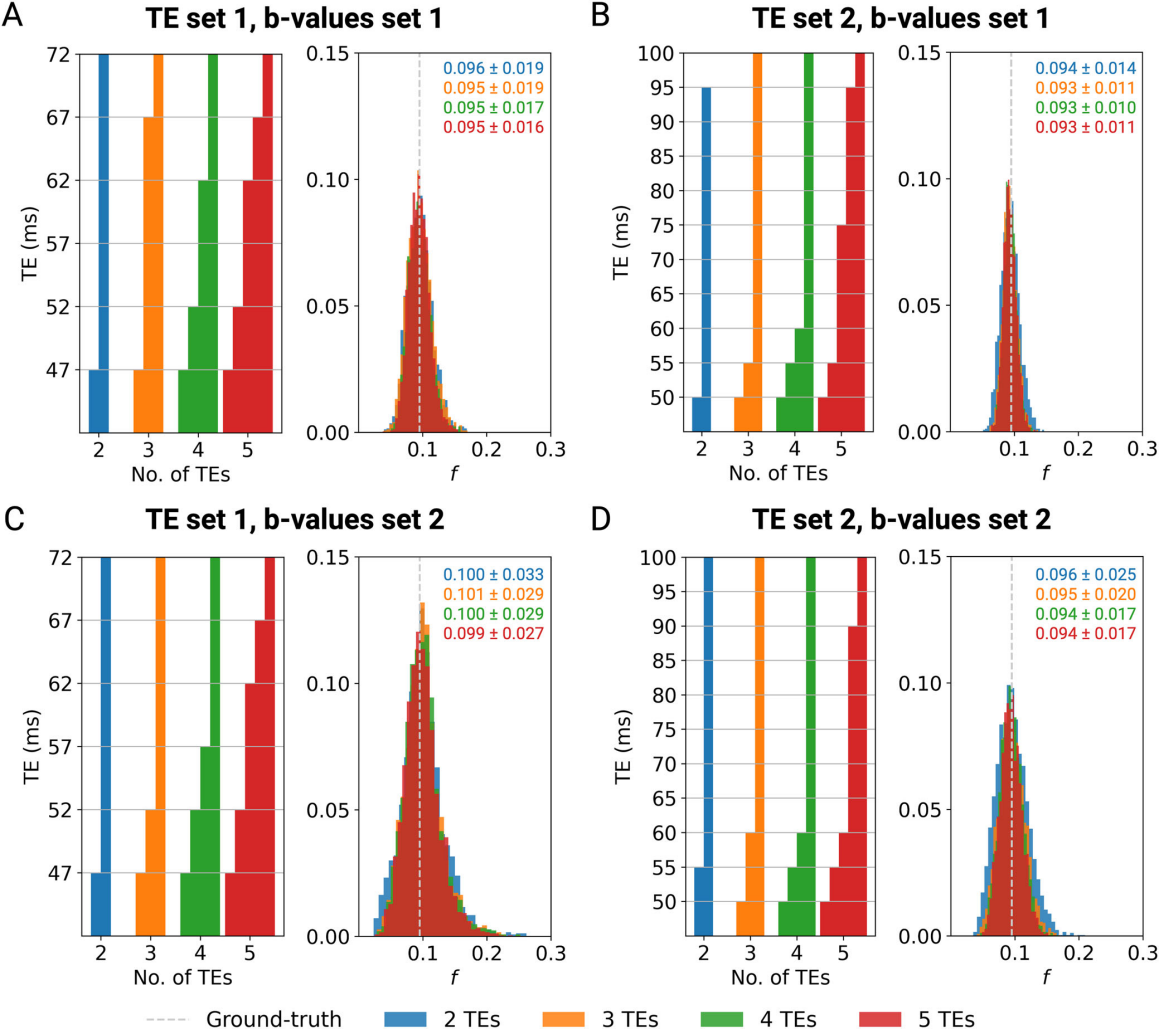
Optimal TEs (left panel) and the corresponding distributions of pseudo‐diffusion volume fraction *f* (right panel) from the 2D T2‐IVIM fit of the liver in silico data (*N*_rep_ = 2500, SNR_S0_ = 40) using these optimal TE values. Four different *b*‐TE protocols were tested: (A) 16 *b*‐values and 6 TE values (*b*‐values set 1 and TE set 1, Table 1); (B) 16 *b*‐values and 11 TE values (*b*‐values set 1 and TE set 2, Table 1); (C) 6 *b*‐values and 6 TE values (*b*‐values set 2 and TE set 1, Table 1); and (D) 6 *b*‐values and 11 TE values (*b*‐values set 2 and TE set 2, Table 1). The protocol (B) yielded the lowest overall variability in f estimation, while the accuracy remained similar across all protocol combinations. The highest bias in f was observed for the protocol (C).

The results of the numerical optimization for the four different combinations of *b*‐values and TE values in the liver are displayed in Figure 6A–D. For each of these combinations, including more TE values in the protocol significantly decreased the variability (i.e., standard deviation, SD) in *f* estimation (SD_5 TE_ < SD_4 TE_ < SD_3 TE_ < SD_2 TE_, *p* < 0.045 for all comparisons). As expected, the overall lowest variability in *f* estimation was achieved for the most extensive protocol (16 *b*‐values, Figure 6B), whereas the accuracy was comparable across all the different combinations with the highest bias in *f* obtained for the shortest protocol (6 *b*‐values, Figure 6C). For the same *b*‐values set, extending the TE range leads to a shift toward higher optimal TE values regardless of the number of TE in the protocol. In general, a protocol with six “consensus” *b*‐values and three TE values (Figure 6D) with (50, 60, and 100 ms) appears to be a suitable compromise between accuracy and scan time. For the “in vivo protocol” (Figure 6A), the optimal three TE values are 47, 67, and 72 ms.

**FIGURE 7:**
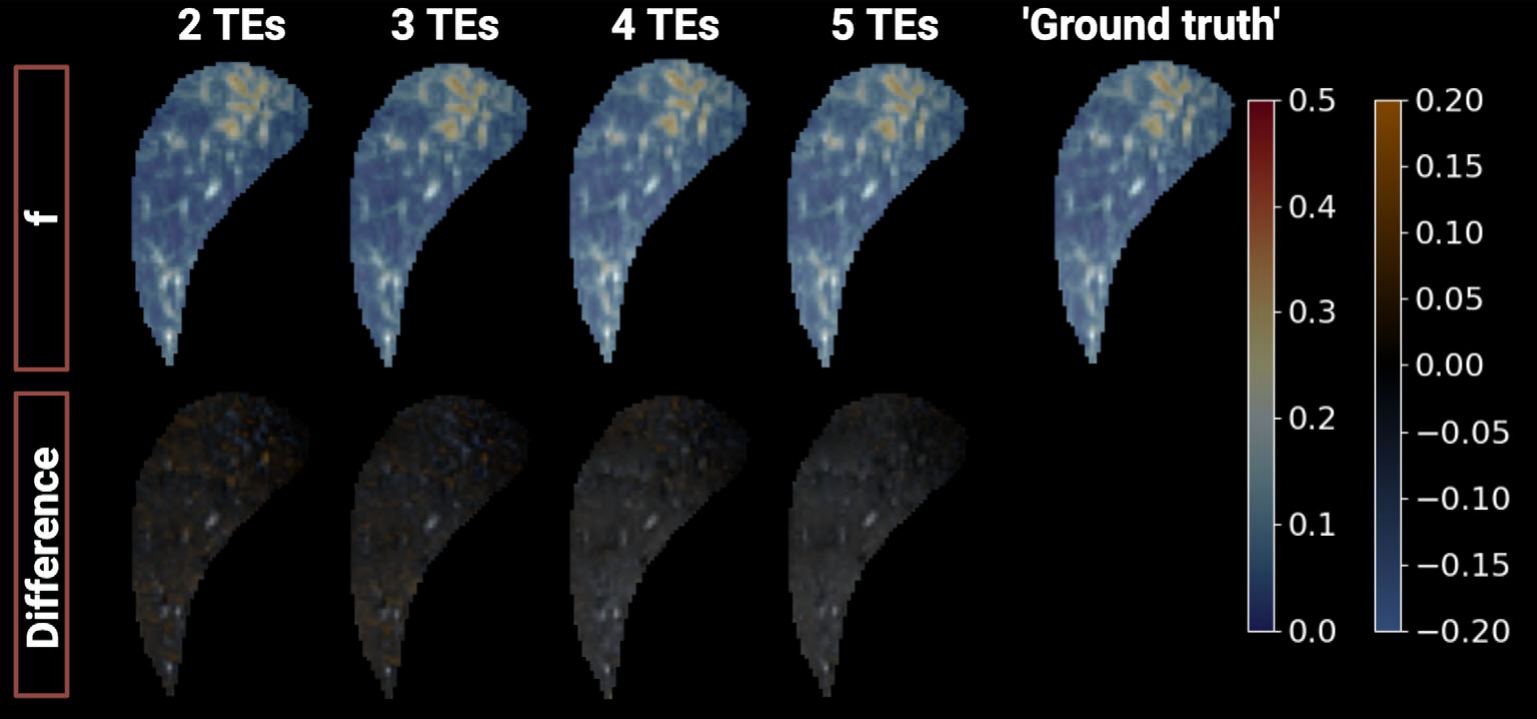
Examples of pseudo‐diffusion volume fraction f maps and corresponding difference maps in the liver of a healthy volunteer. The maps were obtained by fitting the data to the T2‐IVIM model using the optimal TE combinations from TE set 1 and all 16 *b*‐values (Table 1, *b*‐values set 1). The “ground‐truth” *f* map was derived from the 2D T2‐IVIM fit using all 6 TEs and all 16 *b*‐values and was used to compute the difference maps (*f*_true_ − *f*). Similar results were observed for all optimal TE combinations, with slightly overestimated *f* values in regions with higher blood volume fractions relative to the ground‐truth map.

As demonstrated in Figure 7, these findings were validated using a subset of the in vivo data acquired using all 16 *b*‐values and TE set 1. Figure 7A shows the representative *f* maps and the difference maps obtained in the liver, which look very similar for the optimized TE sets.

**FIGURE 8:**
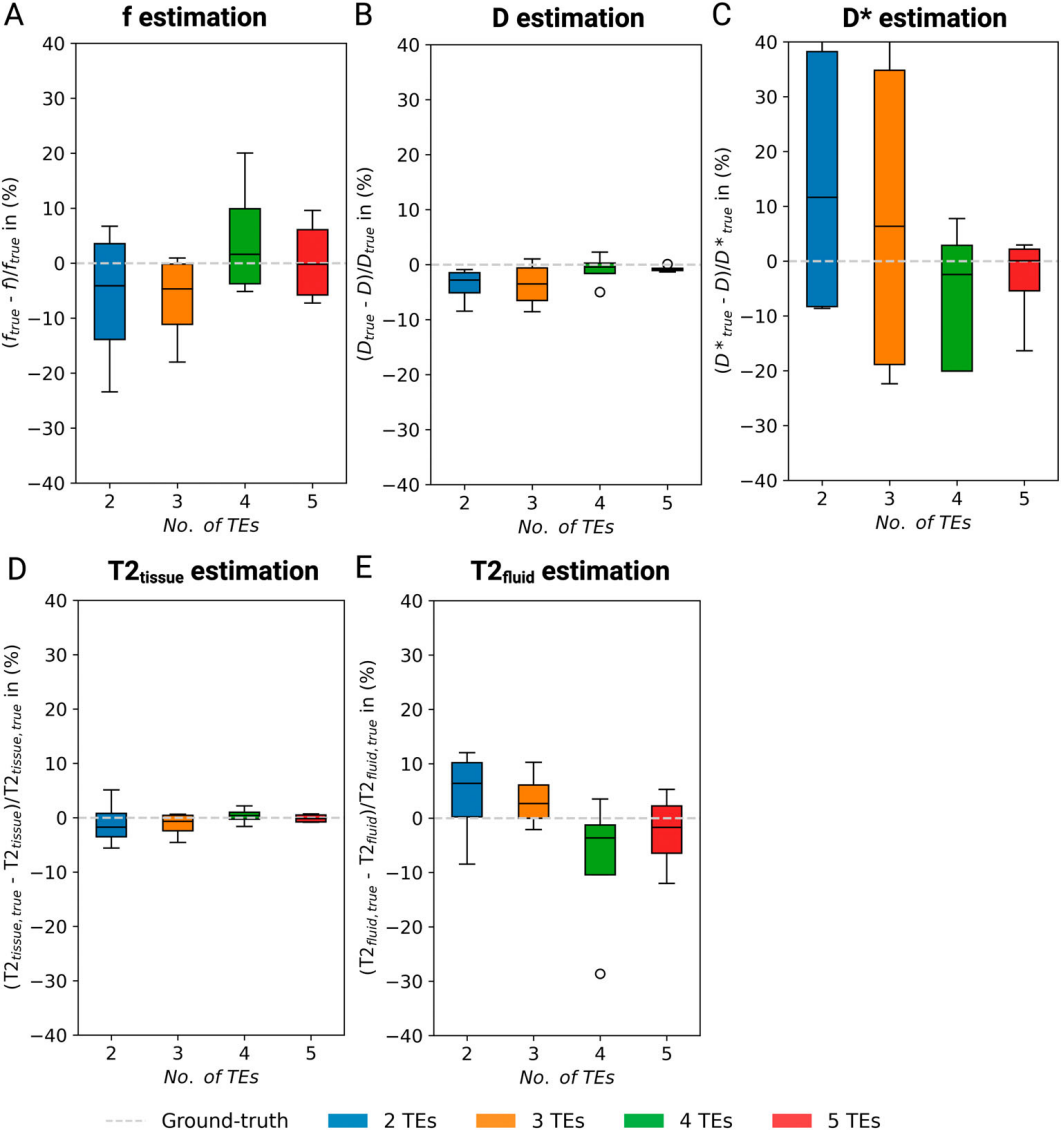
Boxplots of the percent differences between the mean liver “ground truth” values obtained with the in vivo protocol (16 *b*‐values, 6 TE values) and the mean estimates of (A) *f*, (B) *D*, (C) D*, (D) T2_tissue_, and (E) T2_fluid_ obtained with the optimized 2‐, 3‐, 4‐, and 5‐TE protocols in all subjects (*N* = 4). Except for D* estimation, all protocols produced accurate T2 and IVIM parameter estimates (median bias within ±10% of the “ground truth”).

This observation is further confirmed when comparing the percent differences between the mean liver “ground truth” values obtained with the in vivo protocol (16 *b*‐values, 6 TE values) and the mean estimates of all liver pixels for all subjects, as shown in Figure 8. In agreement with the in silico results, the 2D T2‐IVIM approach provides high accuracy in *f* estimation (median bias < 10%), even when only two TE values are used for T2 relaxation correction, compared with the more extensive protocol using six TE values. Note that although the protocol optimization was focused on minimizing the bias and variability of *f*, all protocols enabled relatively accurate estimation of the remaining IVIM and T2 parameters (median bias within ±10%), except for D*.

## 4 Discussion

In this study, we quantitatively assessed bias in pseudo‐diffusion volume fraction when using the traditional IVIM model without accounting for the differences in compartmental T1 and T2 relaxation times. While the T1‐dependence of *f* can be eliminated by choosing long TR, the effect of TE should be minimized by applying simultaneous T2‐IVIM modeling (combined with the IDEAL fitting approach) using two or three different TE values, if possible. Our in vivo findings revealed that the T2‐compensation is particularly important when analyzing the IVIM data collected in the liver.

The evidence from this study supports our hypothesis that the large variability [34, 35] in the reported pseudo‐diffusion volume fractions across different IVIM studies can partially be explained by the variation in TE used for the DW data acquisition at different centers. This variation in TE results from the common practice, both in clinical and research DWI protocols, to use the minimum TE, which is determined by the gradient hardware, maximum *b*‐value and the diffusion encoding scheme. Furthermore, our results also suggest that age‐, physiology‐, or pathology‐related changes in IVIM‐derived *f* cannot straightforwardly be interpreted as changes in blood perfusion or other flow‐related processes unless no changes in compartmental T2 can be assumed. For instance, higher *f* values measured in older subjects maybe be associated with the age‐related increase in liver iron content and thus shorter T2 (contributed by both tissue and blood T2) [36]. In other studies, the extent of reduction in *f* due to perfusion reduction in liver fibrosis could have been overestimated as T2 has also been shown to increase in this pathology [37, 38]. Similarly, as T2 and T2* increase with higher local blood oxygenation [39], *f* is expected to be influenced by the oxygenation status. The use of T2‐IVIM modeling can serve as a potential solution in overcoming these issues by providing TE‐independent *f* measures to improve the reproducibility in multi‐center research settings, and to contribute to better understanding of potentially relevant sources of variation in *f* estimation in various pathologies.

The relaxation time dependence of *f* has been discussed [40, 41] and investigated in vivo in several studies conducted in the normal liver [42, 43, 44], normal prostate [45], pancreas [24], and locally advanced breast cancer [46]. In general, an increase in *f* with longer TE was observed in all these organs, which is consistent with the results of our numerical experiments and in vivo study in the liver. Furthermore, our mean *f* values (between 0.14 ± 0.08 at TE = 47 ms and 0.26 ± 0.10 at TE = 72 ms) measured in the liver are in good agreement with those from previous studies (tab. 4 in Führes et al. [43]). Moreover, the average T2‐corrected *f* estimates obtained in this work (7.88% ± 2.39%) agree well with previously published values obtained with multidimensional IVIM acquisitions at 3T (e.g., 6.43%–8.34% ± 1.86%–1.91% in Simchick et al. [44]; 7.97%–9.35% ± 1.99%–3.06% in Simchick et al. [47]). Additionally, the study by Jerome et al. examining T2‐corrected IVIM in the liver demonstrated that, after accounting for T2 effects and extrapolating to TE = 0, the perfusion fraction in normal liver is on the order of ˜8% (fig. 2b in Jerome et al. [42]), which is consistent with our findings. Similarly, our mean liver T2_fluid_ of 60 ± 6.8 ms is well within the range of values reported for 3 T by Simchik et al. [47] (T2_fluid_ = 1/*R*2_b_ = 55.6 ± 7.6 ms) and at 1.5 T by Jerome et al. [42] (T2_fluid_ = T2_p_ = 77.6 ± 30.2 ms), and notably shorter than the previously reported blood T2 (e.g., 275 ± 50 ms at 3T, with 95% blood oxygenation [23]). As expected, the mean T2_tissue_ of 26.5 ± 2.2 ms value found in our study is slightly lower but overall comparable with the liver T2 values reported by other groups (e.g., 34 ± 4 ms at 3T [48], or 42 ± 3 ms at 3T [23]).

To the best of our knowledge, this is the first study to investigate the TE‐dependence of the IVIM measures in the kidney. We note, however, that TE‐dependence of IVIM parameters has previously been incorporated by Gilani et al. [49] into the velocity model by Wetscherek et al. [50], with the primary focus on renal blood velocity estimation. The relaxation‐compensated *f* values of 0.17 ± 0.03 and 0.13 ± 0.04 measured in the renal cortex and medulla, respectively, are consistent with literature values [2, 25, 27, 51, 52]. The mean T2_tissue_ values (62.5 ± 14.16 and 70.6 ± 21.2 ms in the cortex and medulla, respectively) are close to cortical and medullary T2 values reported by de Bazelaire et al. [48], and the T2_fluid_ values (158.7 ± 58.8 and 154.0 ± 44.1 ms in the cortex and medulla, respectively) are substantially higher than those of the liver. Interestingly, no clear trend in *f* with increasing TE was found in the TE range between 47 and 72 ms. The results of our simulation study appear to confirm these in vivo findings, suggesting that the variability in the *f* estimates may be large and that its TE‐dependence might therefore not be easily detectable in vivo. The lack of TE‐dependence of *f* may also be partially be explained by the additional “pseudo‐diffusion” effect in the kidney caused by the presence of pre‐urine (i.e., glomerular filtrate) flow within the renal tubules (in addition to blood flow in the peritubular capillaries) that introduces a third compartment [53, 54, 55, 56] with distinct relaxation and diffusion properties. In organs such as the kidney, microvascular and tubular flows are known to be highly anisotropic, which challenges the assumption of fully incoherent motion underlying the original pseudo‐diffusion IVIM model and motivates extensions of the framework that explicitly account for directional effects [2, 49, 57, 58, 59]. Furthermore, this pre‐urine compartment is expected to have a relatively long T2 value, which might not have been sufficiently probed at the relatively short TE values used here. This study complement our prior work in which we showed an increase in *f* with increasing diffusion time at constant TE [25]. Future studies with larger sample size, broader range of *b* and TE values, and more advanced T2‐IVIM modeling (e.g., three‐compartment T2‐IVIM modeling) are needed to validate these findings. In addition, future work in the liver should consider multiple fluid compartments, such as portal venous blood and arterial blood, which are known to differ in relaxation rates due to oxygenation differences [39, 60].

In outlook on potential clinical applications, optimizing the T2‐IVIM imaging protocol to ensure the accuracy of the estimated IVIM parameters while minimizing the acquisition time is of particular relevance. For liver T2‐IVIM, Jerome et al. [42] proposed a clinically feasible 11 min protocol at 1.5 T that consisted of one acquisition with 8 *b*‐values (0–800 s/mm^2^) at TE = 62 ms and two additional low *b*‐value scans (0, 10, and 50 s/mm^2^) a two longer TEs (80 and 100 ms). However, this protocol was designed empirically and minimizing the bias in *f* was not the primary goal. Here we propose a numerical framework that optimize the TE values for relaxation‐compensated *f* parameter estimation for any given set of *b*‐values, relaxation properties, and expected IVIM parameters. This flexibility enables efficient protocol optimization for various organs and pathologies if rough estimates of the model parameters are known. Our results suggest that a *b*‐TE protocol consisting of two shorter TEs (50 and 60 ms) and one longer TE (100 ms) combined with six *b*‐values recommended by the recent *consensus* [31] might be optimal for liver T2‐IVIM if the focus is on estimating the pseudo‐diffusion volume fraction. This finding can be explained by the relatively short T2 of the liver (and therefore the need for short TE) and the fact that lower SNR at higher TE values negatively impacts the robustness of the 2D T2‐IVIM modeling. While a minimum TE of 50 ms was used in this study based on the constraints of our imaging system, the optimal T2‐IVIM protocol is inherently hardware‐dependent and may instead include the minimum achievable TE for a given scanner, followed by a second shorter TE and a third longer TE. Nevertheless, the proposed framework and optimization strategy are generalizable and can be adapted to different systems and clinical settings. Further acquisition time reduction could potentially be achieved by allowing variable numbers and values of *b*‐values for each TE in the optimization process, which will be a subject of our future works. In addition to the protocol optimization, the use of accelerated *b*‐TE acquisition methods based on multi‐echo DWI sequences could be considered [61, 62, 63, 64].

Because the pseudo‐diffusion assumption may not always hold in the liver and other abdominal organs, where ballistic or intermediate flow regimes can occur at typical spin‐echo encoding durations, the IVIM signal may also depend on the first‐order motion moment (*M*_1_) [44, 65, 66]. To account for this, several studies have proposed optimized diffusion gradient waveforms designed to strategically sample both *b* and *M*1. More recently, Simchick et al. introduced a *b*‐*M*1‐TE‐optimized acquisition scheme that enables simultaneous and repeatable IVIM and T2 quantification in the liver within a clinically feasible scan time of approximately 6:30 min (with respiratory gating) [47]. In their work, the data sampling strategy was optimized using CLRB analysis based on the TE‐dependent, ballistic IVIM signal model [44, 67]. While this approach provides a compelling alternative to our proposed framework, it is important to recognize that the TEs associated with the optimal *b*‐*M*1‐TE parameters sets are inherently dependent on scanner‐specific hardware and sequence timing constraints.

To date, only one study has investigated whether correcting for TE dependence in IVIM imaging offers added clinical value. Based on their findings in breast cancer patients, Egnell et al. [46] concluded that while the corrected model provides more accurate estimates of *f*, the additional scan time needed to acquire the T2‐IVIM data may not be justified when considering the clinical benefit. One reason for this could be the overall low reliability of the *f* parameter estimation in their study due to low SNR which required fixing the T2_fluid_ (T2_p_) value for fitting. We addressed this issue in our study by applying the IDEAL fitting approach that was shown to be more robust to poor SNR and to provide more robust IVIM parameter estimates than the conventional fitting methods by exploiting the concept of spatial homogeneity [27, 28]. Furthermore, as our method does not require fixing any parameters, it may be better suited for studies investigating the added clinical value of the combined information from the compartmental T2 and IVIM maps. However, if the scan time is a limiting factor, our study suggests that diffusion coefficient *D* might be a better potential biomarker than *f* as it does not depend on TE and its coefficient of variation is typically lower than that of D* [68].

It is worth noting that this study was primarily focused on evaluating the accuracy and robustness of *f* estimation, as there is strong evidence that *f* may represent the most clinically informative IVIM parameter in many liver and kidney applications. In the liver, *f* has been consistently shown to reflect microvascular perfusion changes associated with fibrosis. For instance, a recent review on liver IVIM studies [19] reported that six out of eight studies investigating hepatic fibrosis identified *f* as an effective biomarker, with four demonstrating its superior diagnostic value compared with other IVIM parameters. Similarly, in the kidney—a highly perfused organ—several studies summarized by Caroli et al. [13] have shown that *f* correlates with renal perfusion and with clinical measures of renal function, and in some cases outperforms *D* and D* in tracking disease progression or treatment response.

The main limitations of this study include the small study population, and the limited range of TE values used in the in vivo experiments. Furthermore, the numerical optimization was performed with the sole focus on minimizing the bias and variability of *f* and the determined optimized protocol might not be optimal for other IVIM and T2 parameters. A minor methodological difference between the numerical phantom and in vivo analyses is that *S*_0_ was not estimated in the simulations, as the relaxation‐compensated IVIM equation was explicitly normalized by *S*_0_. This normalization removes this parameter from the fitting process and avoids *S*_0_‐related noise propagation to *f* and the other IVIM and T2 parameters under the controlled simulation conditions. Therefore, omitting *S*_0_ estimation does not affect the assessment of parameter bias. In contrast, in vivo fitting requires explicit estimation of *S*_0_, and associated uncertainty may propagate to coupled IVIM parameters. More broadly, the absence of in vivo “ground truth” and test–retest data further limits our ability to directly evaluate the accuracy and precision of the estimated T2 and IVIM parameters using the proposed method. This will be pursued in prospective studies in which the proposed technique and the optimized protocol will be applied in a range of hepatic pathologies.

To summarize, this study provides a systematic evaluation of the TE dependence of IVIM parameters, with a particular focus on pseudo‐diffusion volume fraction, in both the liver and kidney. It also offers practical guidelines for future studies, including potential modifications to existing recommendations [35] and guidance on TE selection for liver acquisitions, analogous to approaches used for *b*‐value optimization [69].

## 5 Conclusion

Based on simulation results, differences in T2 between tissue and fluid compartments can introduce a TE‐dependent bias in the pseudo‐diffusion volume fraction with the traditional IVIM model. Our study makes several novel contributions: it quantifies this effect, introduces a 2D IDEAL fitting approach for simultaneous estimation of IVIM parameters and compartmental T2 values, and provides a framework for optimizing *b*‐TE protocols. Using this approach, we show that the TE‐related bias in *f* can be effectively corrected in the liver. In the kidney, however, no TE‐dependence of IVIM parameters was observed in vivo, suggesting that such corrections may be organ‐specific and emphasizing the need to evaluate TE effects individually for each tissue type.

## Funding

This work was supported by the National Institutes of Health (K99 DK138294, K99 AG080084) and the Deutsche Forschungsgemeinschaft (408765040, 497764939).

## Conflicts of Interest

The authors declare no conflicts of interest.

## Supporting information


**Figure S1:** A contour plot of bias in pseudo‐diffusion volume fraction *f* (%) calculated using simulated kidney data (Table 1, TE set 1 and *b*‐values set 1) as a function of differences in compartmental relaxation times (ΔT1 = T1_fluid_ − T1_tissue_ and ΔT2 = T2_fluid_ − T2_tissue_) at various TE (52, 62, and 72 ms) and TR (2000, 3000, and 4000 ms) generated without Rician noise. The bias was calculated as 100 × (*f*_fit_ − *f*_true_)/*f*_true_. While the effect of ΔT1 on *f* parameter estimation is minimal, ΔT2 > 20 ms leads to < 20% bias at TE = 52 ms and > 20% bias at TE = 72 ms.
**Figure S2:** Distributions of pseudo‐diffusion volume fraction *f* obtained from 2D T2‐IVIM modeling (blue) and conventional IVIM modeling at TE = 52 ms (yellow), TE = 62 ms (green), and TE = 72 ms (red) using the simulated kidney data (Table 1, TE set 1, *b*‐values set 1) for various ΔT1 = T1_fluid_ − T1_tissue_ and ΔT2 = T2_fluid_ − T2_tissue_. The gray dotted line represents the “ground truth” *f*_true_ value. Monte‐Carlo simulations were performed with *N*_rep_ = 2500 repetitions and SNR_S0_ = 40. The TR was kept constant at 4000 ms. The 2D T2‐IVIM fitting led to lower variability, as reflected by significantly lower standard deviation (*p* < 0.0001 for all comparisons). Mean ± SD across 2500 repetitions is reported for each distribution.
**Figure S3:** Distributions of pseudo‐diffusion volume fraction *f* obtained from 2D T2‐IVIM modeling (blue) and conventional IVIM modeling at TE = 52 ms (yellow), TE = 62 ms (green), and TE = 72 ms (red) using the simulated liver data (Table 1, TE set 1, *b*‐values set 1) for various SNR levels (33–200) for ΔT1 = T1_fluid_ − T1_tissue_ = 300 ms and ΔT2 = T2_fluid_ − T2_tissue_ = 15 ms. The gray dotted line represents the “ground truth” *f*_true_ value. Monte‐Carlo simulations were performed with *N*_rep_ = 2500 repetitions and SNR_S0_ = 40. The TR was kept constant at 4000 ms. 2D T2‐IVIM fitting provided accurate f estimates over a wide range of SNR levels. Although the variability of the estimates increased at lower SNR, the standard deviation of the distributions from the 2D fit was significantly lower than that from the conventional 1D IVIM fit (*p* < 0.0001 for all comparisons).
**Figure S4:** Percentage bias in (A) diffusion coefficient *D*, (B) pseudo‐diffusion coefficient D* and (C) pseudo‐diffusion volume fraction *f*, obtained from 2D T2‐IVIM modeling (blue) and conventional IVIM modeling at TE = 52 ms (yellow), TE = 62 ms (green), and TE = 72 ms (red), using simulated liver data (Table 1, TE set 1, *b*‐value set 1) for various SNR levels (33–200), with ΔT1 = T1_fluid_ − T1_tissue_ = 300 ms and ΔT2 = T2_fluid_ − T2_tissue_ = 15 ms. Bias was calculated as 100 × (*θ*_fit_ − *θ*_true_)∖*θ*_true_, where *θ* denotes the parameter of interest. The addition of noise increased the variability of *f* estimates across all methods without affecting accuracy. In contrast, D* showed reduced accuracy and increased variability at lower SNR for the conventional IVIM model, but not for the 2D T2‐IVIM model.

## Data Availability

Data analysis scripts used in this study are available on Github: https://github.com/stabinska/T2‐IVIM.
