# Supplementary material for: Toward Optimized Intravoxel Incoherent Motion (IVIM) and Compartmental T2 Mapping in Abdominal Organs
Source: Magn Reson Med. 2026 Feb 1;95(6):3161–75. doi: 10.1002/mrm.70278 (PMC12922574; doi:10.1002/mrm.70278)
Supplement: Supplementary file 1 — Figure S1: A contour plot of bias in pseudo‐diffusion volume fraction f (%) calculated using simulated kidney data (Table 1, TE set 1 and b‐values set 1) as a function of differences in compartmental relaxation times (ΔT1 = T1fluid − T1tissue and ΔT2 = T2fluid − T2tissue) at various TE (52, 62, and 72 ms) and TR (2000, 3000, and 4000 ms) generated without Rician noise. The bias was calculated as 100 × (f fit − f true)/f true. While the effect of ΔT1 on f parameter estimation is minimal, ΔT2 > 20 ms leads to < 20% bias at TE = 52 ms and > 20% bias at TE = 72 ms. Figure S2: Distributions of pseudo‐diffusion volume fraction f obtained from 2D T2‐IVIM modeling (blue) and conventional IVIM modeling at TE = 52 ms (yellow), TE = 62 ms (green), and TE = 72 ms (red) using the simulated kidney data (Table 1, TE set 1, b‐values set 1) for various ΔT1 = T1fluid − T1tissue and ΔT2 = T2fluid − T2tissue. The gray dotted line represents the “ground truth” f true value. Monte‐Carlo simulations were performed with N rep = 2500 repetitions and SNRS0 = 40. The TR was kept constant at 4000 ms. The 2D T2‐IVIM fitting led to lower variability, as reflected by significantly lower standard deviation (p < 0.0001 for all comparisons). Mean ± SD across 2500 repetitions is reported for each distribution. Figure S3: Distributions of pseudo‐diffusion volume fraction f obtained from 2D T2‐IVIM modeling (blue) and conventional IVIM modeling at TE = 52 ms (yellow), TE = 62 ms (green), and TE = 72 ms (red) using the simulated liver data (Table 1, TE set 1, b‐values set 1) for various SNR levels (33–200) for ΔT1 = T1fluid − T1tissue = 300 ms and ΔT2 = T2fluid − T2tissue = 15 ms. The gray dotted line represents the “ground truth” f true value. Monte‐Carlo simulations were performed with N rep = 2500 repetitions and SNRS0 = 40. The TR was kept constant at 4000 ms. 2D T2‐IVIM fitting provided accurate f estimates over a wide range of SNR levels. Although the variability of the estimates increased at lowe [file MRM-95-3161-s001.docx]

**Supporting Information**


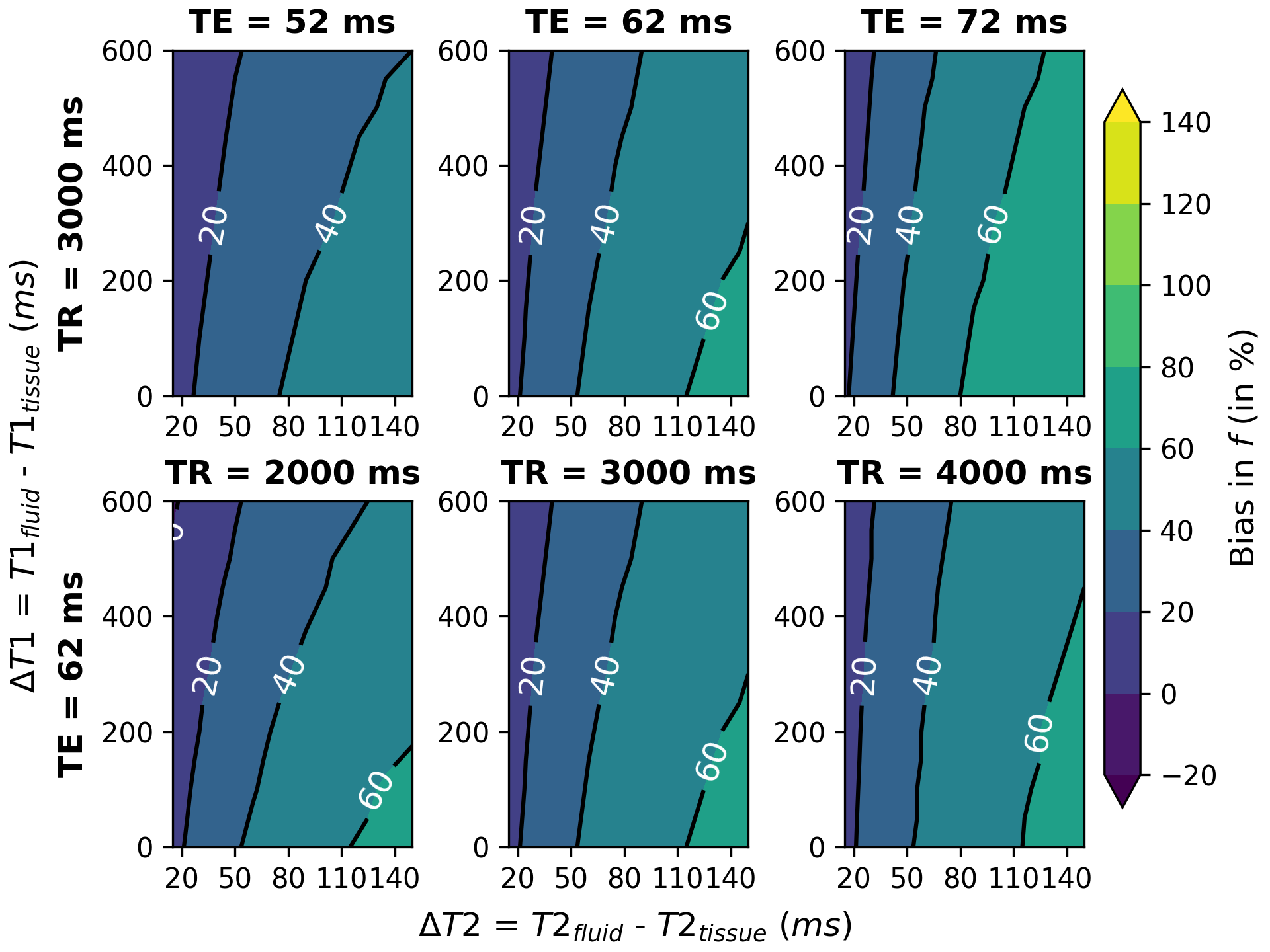


**Figure S1:** A contour plot of bias in pseudo-diffusion volume fraction f (%) calculated using simulated kidney data (Table 1, TE set 1 and b-values set 1) as a function of differences in compartmental relaxation times (ΔT1 = T1_fluid_ – T1_tissue_ and ΔT2 = T2_fluid_ – T2_tissue_) at various TE (52 ms, 62 ms, and 72 ms) and TR (2000 ms, 3000 ms, and 4000 ms) generated without Rician noise. The bias was calculated as 100×(f_fit_ – f_true_)/f_true_. While the effect of ΔT1 on f parameter estimation is minimal, ΔT2 > 20 ms leads to <20% bias at TE = 52 ms and >20% bias at TE = 72 ms.


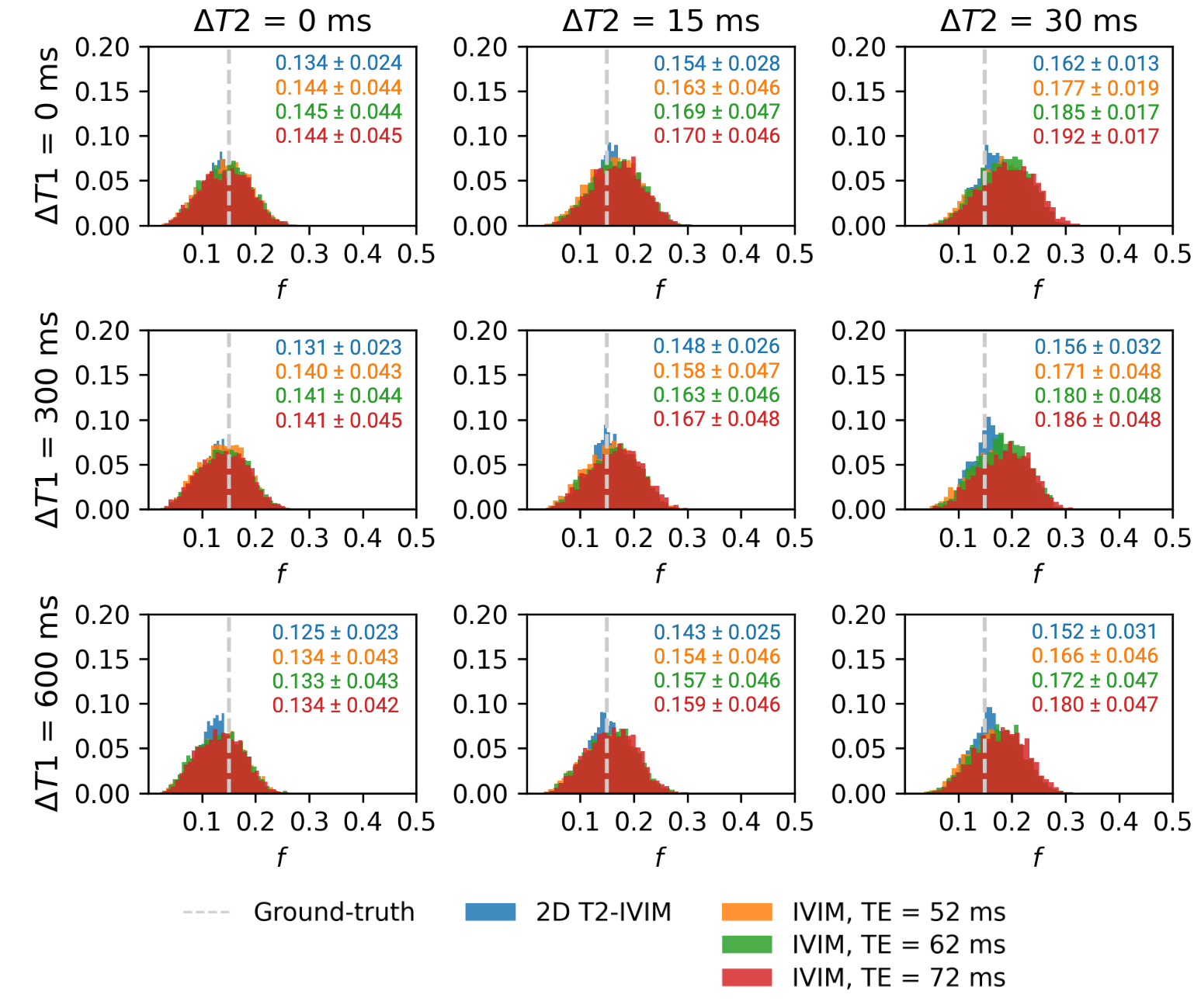


**Figure S2:** Distributions of pseudo-diffusion volume fraction f obtained from 2D T2-IVIM modeling (blue) and conventional IVIM modeling at TE = 52 ms (yellow), TE = 62 ms (green), and TE = 72 ms (red) using the simulated kidney data (Table 1, TE set 1, b-values set 1) for various ΔT1 = T1_fluid_ – T1_tissue_ and ΔT2 = T2_fluid_ – T2_tissue_. The grey dotted line represents the ‘ground truth’ f_true_ value. Monte-Carlo simulations were performed with N_rep_ = 2500 repetitions and SNR_S0_ = 40. The TR was kept constant at 4000 ms. The 2D T2-IVIM fitting led to lower variability, as reflected by significantly lower standard deviation (P < 0.0001 for all comparisons). Mean ± SD across 2500 repetitions is reported for each distribution.


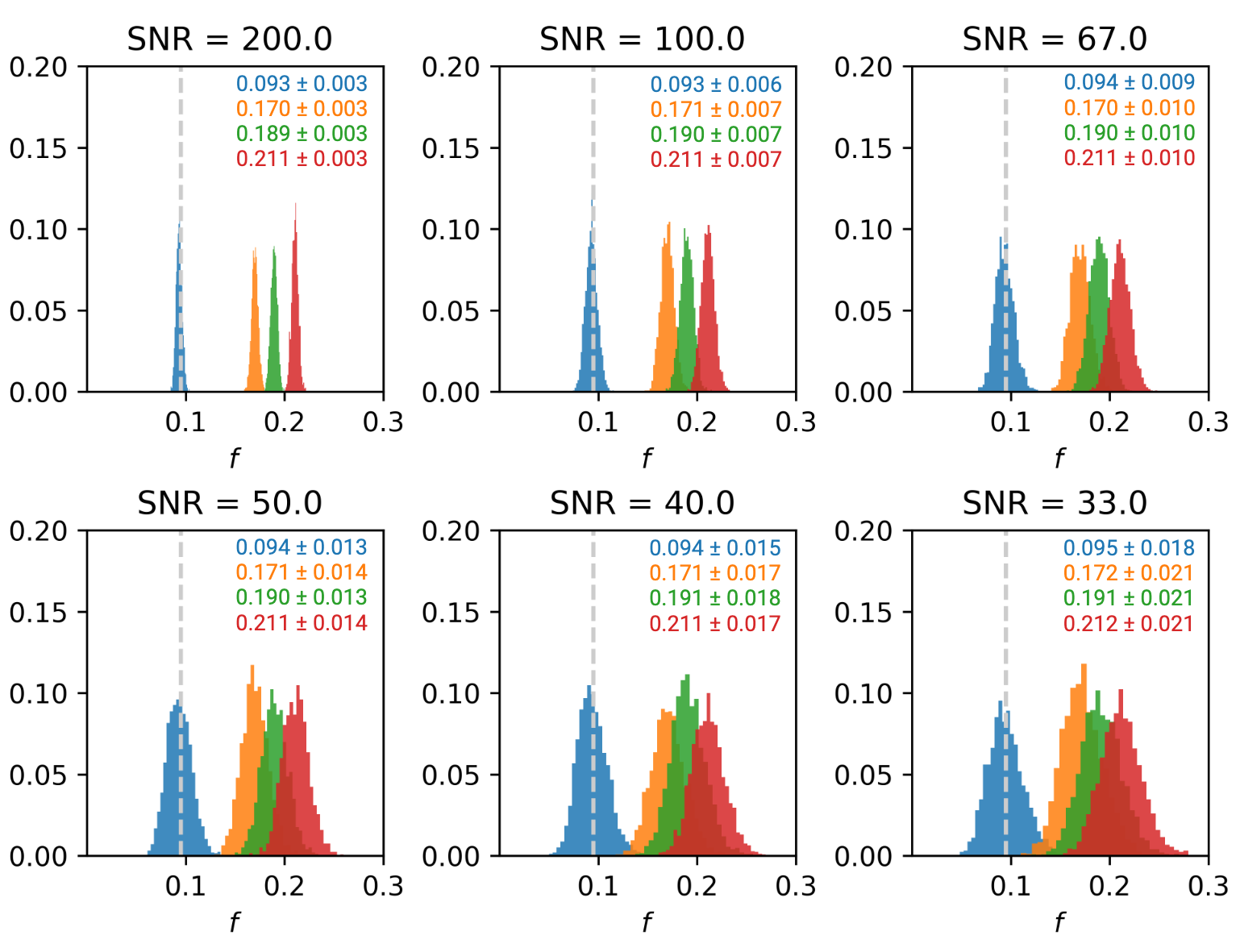


**Figure S3:** Distributions of pseudo-diffusion volume fraction f obtained from 2D T2-IVIM modeling (blue) and conventional IVIM modeling at TE = 52 ms (yellow), TE = 62 ms (green), and TE = 72 ms (red) using the simulated liver data (Table 1, TE set 1, b-values set 1) for various SNR levels (33 – 200) for ΔT1 = T1_fluid_ – T1_tissue_ = 300 ms and ΔT2 = T2_fluid_ – T2_tissue_ = 15 ms. The grey dotted line represents the ‘ground truth’ f_true_ value. Monte-Carlo simulations were performed with N_rep_ = 2500 repetitions and SNR_S0_ = 40. The TR was kept constant at 4000 ms. 2D T2-IVIM fitting provided accurate f estimates over a wide range of SNR levels. Although the variability of the estimates increased at lower SNR, the standard deviation of the distributions from the 2D fit was significantly lower than that from the conventional 1D IVIM fit (P < 0.0001 for all comparisons).


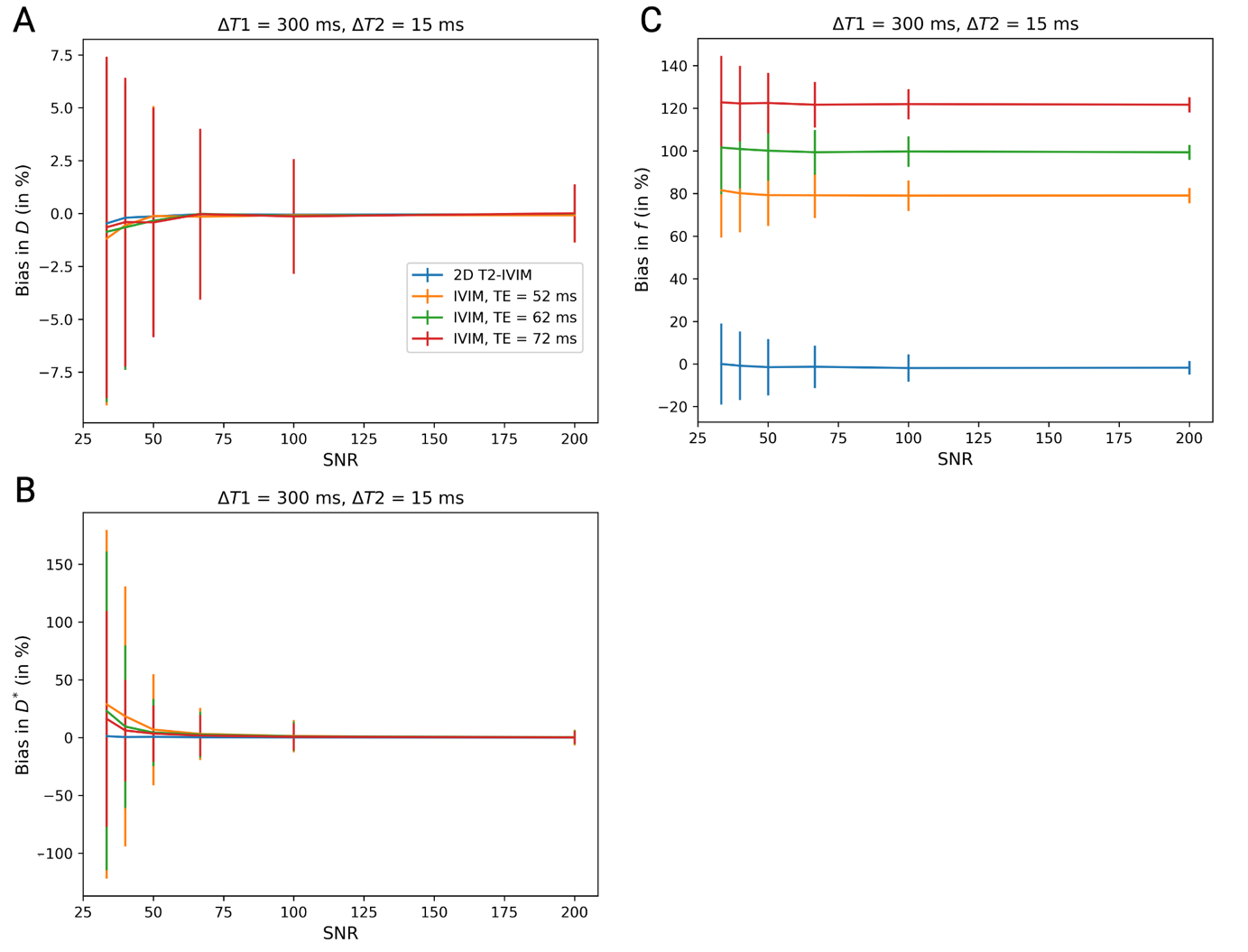


**Figure S4:** Percentage bias in (A) diffusion coefficient D, (B) pseudo-diffusion coefficient D* and (C) pseudo-diffusion volume fraction f, obtained from 2D T2–IVIM modeling (blue) and conventional IVIM modeling at TE = 52 ms (yellow), TE = 62 ms (green), and TE = 72 ms (red), using simulated liver data (Table 1, TE set 1, b-value set 1) for various SNR levels (33–200), with ΔT1 = T1_fluid_ − T1_tissue​_ = 300 ms and ΔT2 = T2_fluid_ − T2_tissue_​ = 15 ms. Bias was calculated as 100 × (θ_fit_ – θ_true_)\θ_true_ where θ denotes the parameter of interest. The addition of noise increased the variability of f estimates across all methods without affecting accuracy. In contrast, D* showed reduced accuracy and increased variability at lower SNR for the conventional IVIM model, but not for the 2D T2–IVIM model.
